# Plasma-induced unconventional shock waves on oil surfaces

**DOI:** 10.1038/s41598-018-36278-3

**Published:** 2018-12-13

**Authors:** Guoliang Li, Ruiheng Hu, Jau Tang, Huai Zheng, Sheng Liu

**Affiliations:** 1 Institute of Technological Sciences, Wuhan University, Wuhan, Hubei 430072 China; 2 Arthur Amos Noyes Laboratory of Chemical Physics, California Institute of Technology, Pasadena, CA 91125 USA; 3 School of Power and Mechanical Engineering, Wuhan University, Wuhan, Hubei 430072 China

## Abstract

Electric corona discharge in a multi-phase system results in complex electro-hydrodynamic phenomena. We observed unconventional shock wave propagation on an oil thin film sprayed over a polymer-coated conductor. A hair-thin single shock wave arose when the high voltage bias of an overhung steel needle was abruptly removed. However, such solitary waves possess neither interference nor reflection properties commonly known for ordinary waves, and also differ from the solitons in a canal or an optical fiber. We also observed time-retarded movement for dispersed oil droplets at various distances from the epicenter which have no physical contact, as if a wave propagating on a continuous medium. Such a causality phenomenon for noncontact droplets to move resembling wave propagation could not be possibly described by the conventional surface wave equation. Our systematic studies reveal a mechanism involving oil surface charges driven by reminiscent electric fields in the air when the needle bias is suddenly removed.

## Introduction

Plasma technology has found wide applications, covering energy, environment and health related aspects of life, such as plasma lighting^1,2^, plasma display^3^, IC wafer treatment^4^, plasma gasification^5^, plasma waste processing^6^, food engineering^7^ and many medical applications^8^. It is very important to better understand the interactions among materials of different phases, such as gas, liquid, solid as well as plasma in the presence of a very strong electric field. Because the details about the mutual interactions among different components are not fully understood, integration of electrodynamics, hydrodynamics, aerodynamics and fluid dynamics for a multi-phase system is complicated and remains to be a challenging subject for investigation. Among the many instances of interacting multi-phase system, corona discharge, for example, has wide applications, including photocopying, initiation of electrochemical processes, oil-water separation, waste treatment, etc.

In this letter we focus on particularly the dynamic wave-like behavior of an oil thin film or oil droplets induced by electric corona. From such experiments we could explore the electro-hydrodynamics and fluid dynamics of a composite system of liquid, air, plasma and a conductor. In one experiment, an oil film or several oil droplets were placed on top of a polymer-coated conducting ITO (indium tin oxide) glass. We report here the observation of a hair-thin shock wave without ripples on the oil surface when the bias voltage was abruptly removed from a steel needle directly placed above the film. In this work we call such a single shock wave a solitary wave to distinguish from the conventional solitons. Systematic studies of how environmental changes affect the shock wave propagation are also presented. This work unravels some exotic wave behaviors and elucidates the underlying mechanism. Such a solitary wave behaves differently from the earthquake tsunami wave^9^ and also characteristically differently from the soliton wave in a canal or optical fiber^10,11^.

It is well-known that solitons in canals or optical fibers arise as a result of cancellation of nonlinearity and dispersion of the propagation media. However, the hair-thin solitary wave we reported in the work does not have ripples as water waves. Moreover, such non-conventional shock waves do not possess the commonly known wave features such as reflection and interference. We have discovered that the driving force for the solitary shock wave to propagate does not come from the oil itself. Instead, we found that the wave propagation is caused by the Coulomb forces between the surface charged oil molecules and the electric corona^12^ induced by a sharp steel needle with a high voltage bias. Electric corona discharge and associated dynamics have been an area of intensive research with many applications in semiconductor wafer processes and waste treatment as mentioned earlier, but also in oil-water separation^13^, fluid pumping^14^ and heat transfer^15^, etc. Unlike the common waves that could be described by the well-known wave equation, these peculiar solitary wave phenomena reported here are different in many aspects from the conventional waves, soliton or even tsunami waves.

## Results

**Figure 1:**
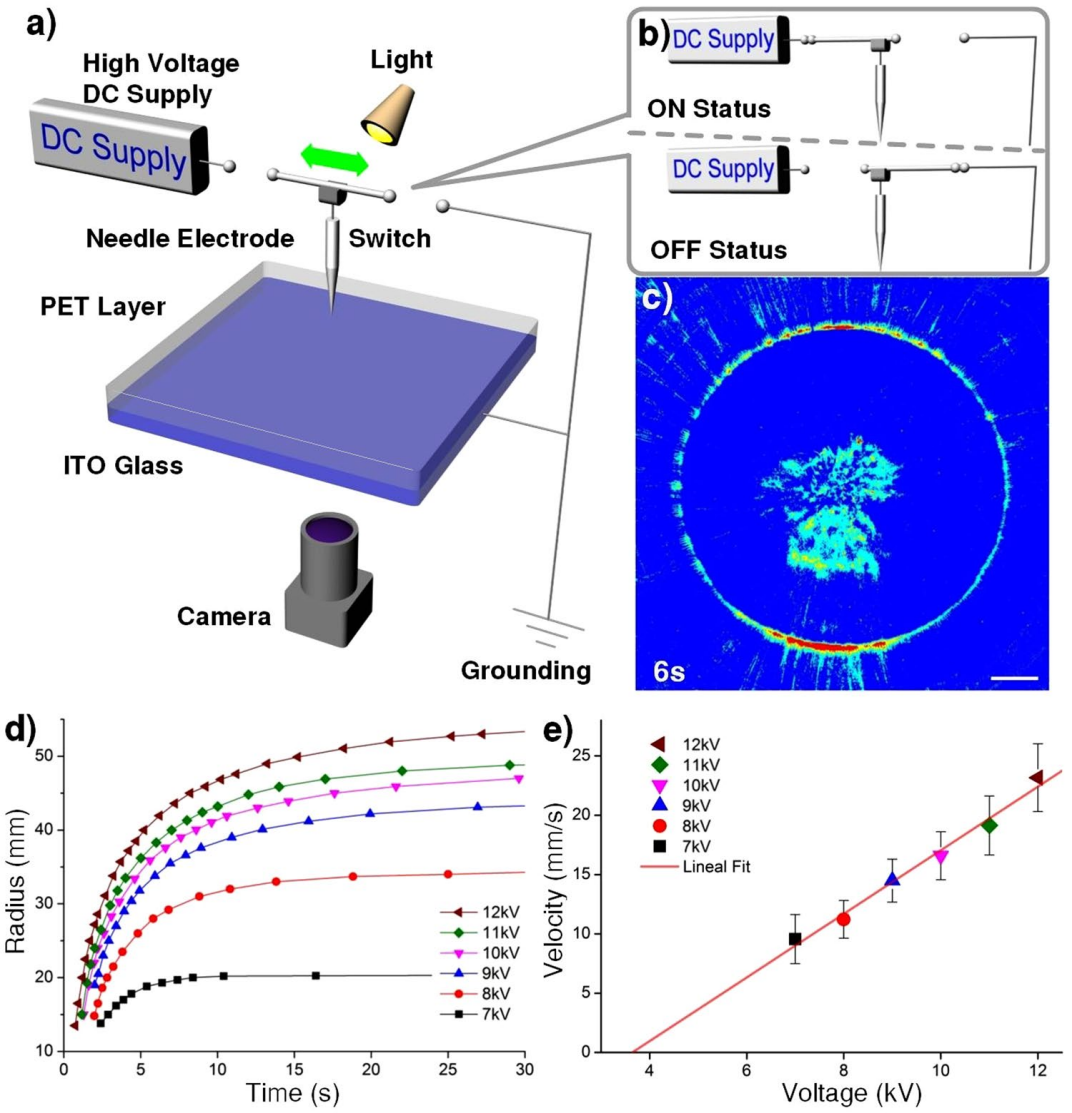
Schematic illustration of the setup and solitary shock wave propagation. (**a**) Schematic diagram for the setup, showing a steel needle, biased by a high voltage. **(b**, top**)** Schematic diagram for the on-status for the setup, showing a steel needle biased by a high voltage DC power supply. (**b**, bottom) Similar diagram for the off-status except that the bias voltage is abruptly removed and the needle is grounded simultaneously to induce a solitary shock wave. (**c)** Photo images at 5 s showing the propagation of a hair-thin solitary shock wave from the epicenter directly below the needle. Original black and white photo images from the camera were color-coded to improve visual contrast. A DC voltage of 8 kV and a gap height of 15 mm between the needle and the oil film were used. (**d)** The time dependence of the ring radius of the solitary wave at various DC voltage biases in the range of 7-12 kV. A gap height of 15 mm was used. The wave travelling distance appears to follow a bi-exponential attenuation curve, indicating slowing down at long times. A positive voltage bias was used. The error bars are too small to be shown. (**e)** The corresponding initial wave velocity versus DC voltage bias, showing a linear dependence and a threshold voltage of about 4 kV for the solitary wave to occur. By fitting of curves in the top figure using a bi-exponential function and then calculating the first time derivative one can obtain the initial wave velocity.

To substantiate our claims we have performed systematic studies to investigate how the environmental changes affect the wave formation and propagation. The schematic diagram for the apparatus to observe and to study solitary shock waves on an oil surface are illustrated in Fig. 1a,b. A video camera was placed directly under the ITO substrate. To abruptly disconnect the steel needle from the high voltage DC power supply and also to simultaneously ground the needle, we installed a switch between the DC power supply and the needle, and also between the needle and the ground. During the experiments we first charge the steel needle to 8 kV, a shock wave was found to travel outward and we waited for the oil surface to become stabilized after about 5 s. Then, we abruptly removed the bias voltage using a switch and connected the needle to the electric ground, as shown in Fig. 1c we observed a hair-thin, ring-shaped solitary wave, like a soliton or a single tsunami wave but with no ripples, traveling outward from the epicenter directly underneath the needle (see Supplementary Video 1). The bar for all figures represent 10 mm in length. The initial shock wave caused by needle charging is different from the solitary shock wave upon needle ground termination, and only the later could produce hair-thin ring-shaped shock wave. Figure 1d illustrates the time dependence of the ring radius of the solitary wave at several DC voltage biases. The time dependent profiles could be fitted well by a bi-exponential function empirically to extract the corresponding wave velocity at short times. As shown in Fig. 1e, a simple linear dependence of the initial speed on the bias was obtained, but with a threshold voltage for the wave behavior to appear. The leveling-off for the travel distance indicates the wave speed decreases in time. In contrast, solitons in a canal or an optical fibers propagate at a constant speed and could travel a long distance.

**Figure 2:**
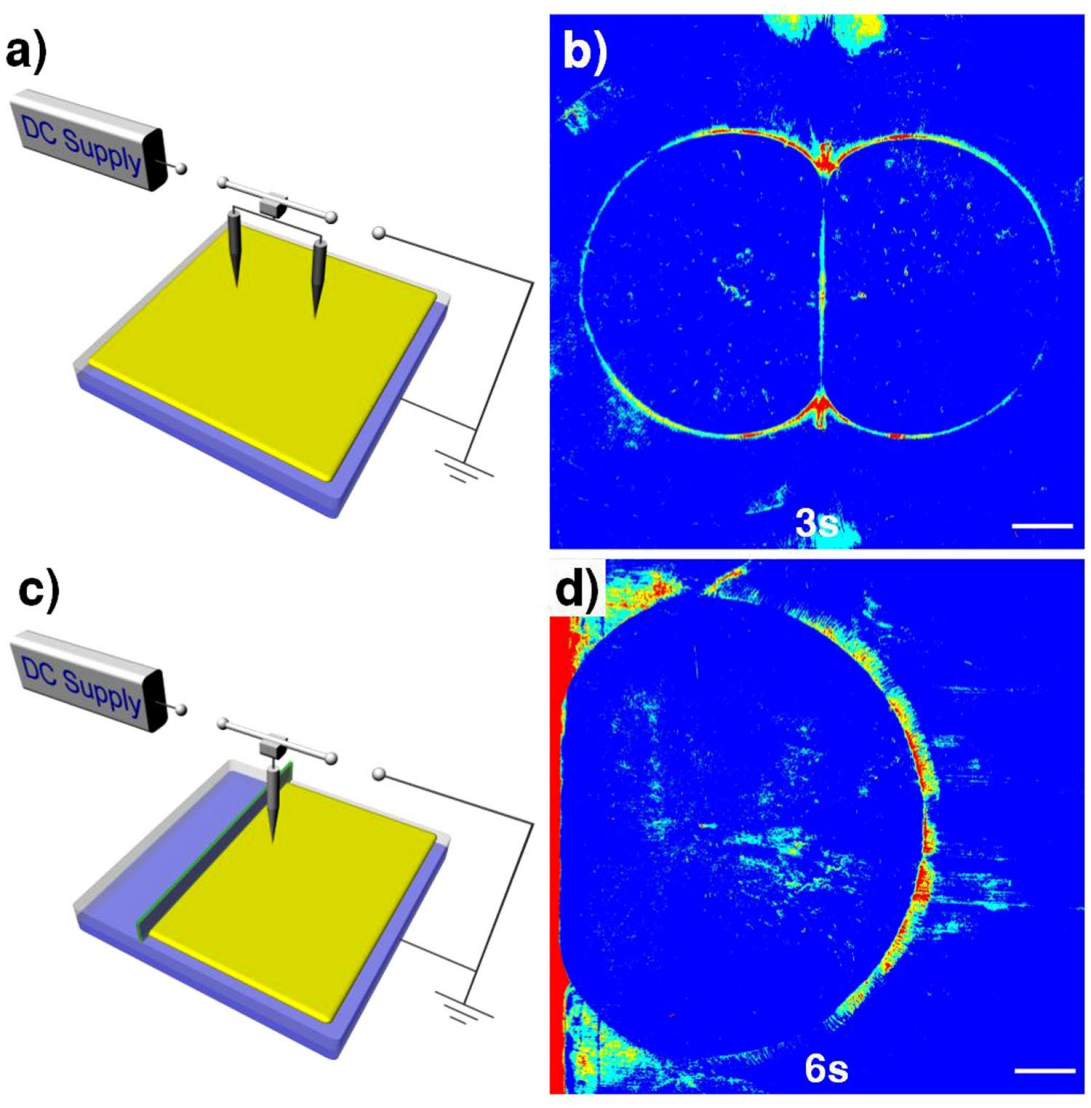
Unconventional solitary wave propagation. (**a**) The schematic illustration for the setup for the experiment involving two shock waves from a pair of parallel needles at a separation of 48 mm and above the polymer film of 15 mm. After the DC bias voltage reached 9 kV, then after 5 s the switch was shut off. (**b)** Photo image of two solitary wave fronts after collision. Unlike conventional colliding solitons, two wave fronts formed a joint midway boundary. **(c)** Schematic setup illustration to demonstrate absence of boundary reflection of the shock wave. A long narrow strip of a PET film of 120 mm × 9 mm × 0.1 mm was placed as a short wall at a distance from the epicenter of 23 mm, and then was glued onto the PET-coated ITO using a resin glue (EVA resin). The needle gap height was 15 mm ad the bias voltage was 9 kV. **(d)** Photo mage of a solitary shock wave after hitting a short wall near the left frame producing no reflected waves at all, unlike conventional surface waves or solitons.

Illustrated in Fig. 2a,b are the schematic diagram for setup and the photo image of two colliding solitary wave fronts (see Supplementary Video 2). It is well-known that two colliding solitons should pass each other upon collision. However, two solitary waves here could not pass through each other but rather formed a joint midway boundary. In another counter example for this unconventional solitary wave, as illustrated in Fig. 2c,d, no wave reflection was observed after hitting a short wall near the left frame (see Supplementary Video 3).

**Figure 3:**
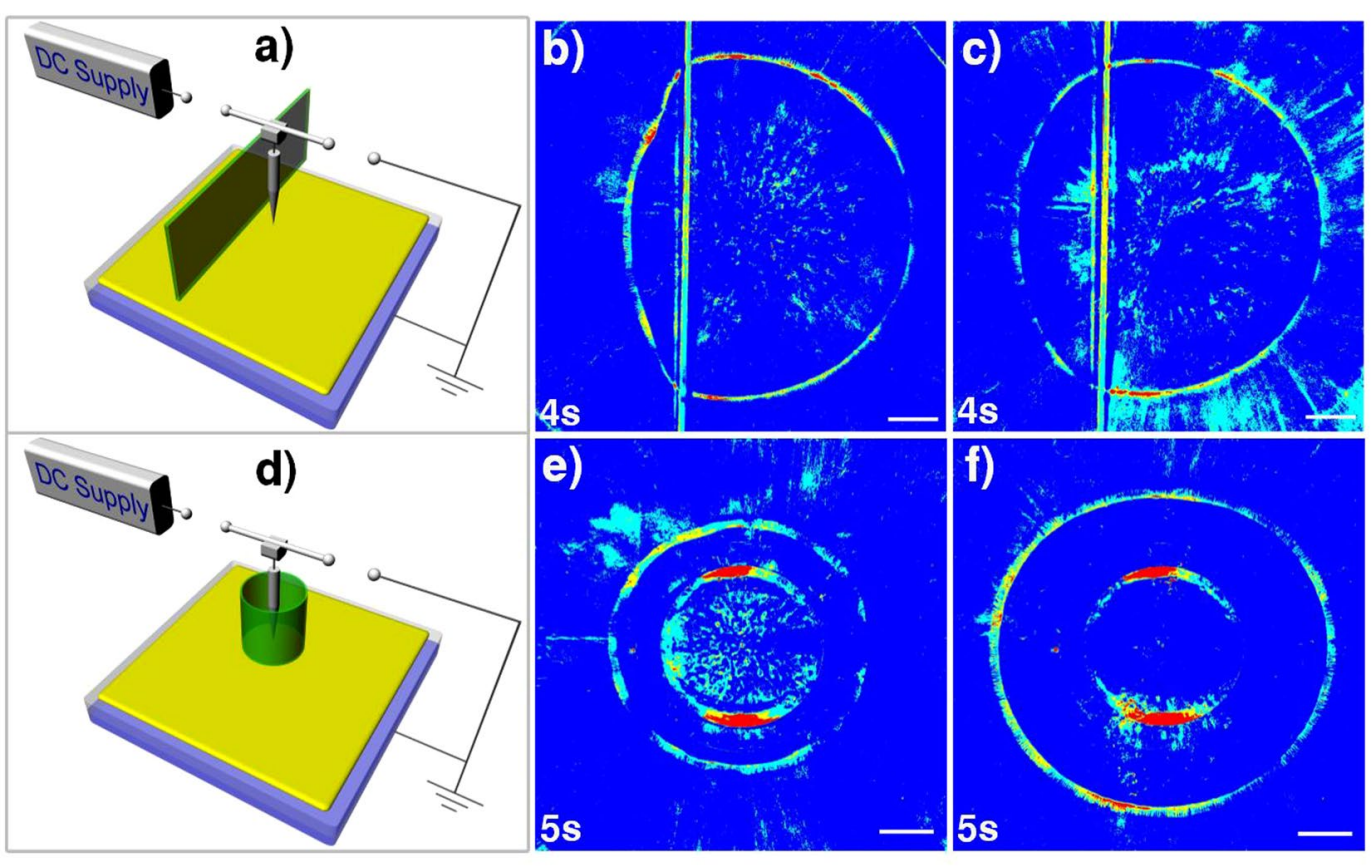
Distorted solitary shock waves due to an obstructing object in the air. (**a**) Schematic setup diagram for the experiments involving a **t**all vertical acrylic wall of a dimension of 110 mm × 50 mm × 1.2 mm. This wall is placed at 11 mm away from the needle. During the ON status the distance between the lower edge of this acrylic plate was placed at 20 mm above the polymer film surface, whereas during the OFF status before shutting off the power, the gap between the lower edge of this acrylic plate was changed to 4 and 10 mm, respectively, for comparison. The needle gap height was kept at 15 mm. (**b**,**c**) Effects on wave propagation due to a vertical plate placed 4 and 10 mm, respectively, over the left half plane of the oil film. These insulation half plates could significantly affect wave propagation on the left hand side of the oil film. (**d**) The schematic setup diagram for the experiments involving enclosure of the needle at the center by an acrylic cylinder of a length of 50 mm and an inner diameter of 30 mm and an outer diameter of 39 mm. During the ON status the tube was placed at 20 mm above the polymer film surface, whereas during OFF status the power was shut off. Two different tube heights of 4 and 10 mm were used for comparison. The needle gap height was kept at 15 mm and the bias voltage was 9 kV. (**e**,**f**) Constrained wave propagation for a needle surrounded by an acrylic tube of a radius of 15 mm but with a different height of 4 and 10 mm, respectively, between the tube and the oil film. The long-time ring size of the confined solitary wave increases with the gap height.

Moreover, we have performed a series of tests to elucidate the mechanisms. Figure 3a–f exhibit distortion of the shock wave due to an obstructing object in the air. Shown in Fig. 3a–c are the images for the solitary shock wave propagation when an insulating plate was placed vertically over the left half plane of the oil film, showing influences on wave propagation on the left hand side of the oil film (see Supplementary Video 4). Figure 3d–f represent shock wave propagation with a needle surrounded at the center by a cylindrical acrylic tube which was placed at a height of 4 and 10 mm, respectively, above the polymer layer surface (see Supplementary Video 5). At a 4 mm gap height, the ring-shaped shock wave propagation was pretty much confined by the tube, whereas at a 10 mm gap height the final ring radius could exceed beyond the tube radius, but still far smaller than the case without a confining tube.

**Figure 4:**
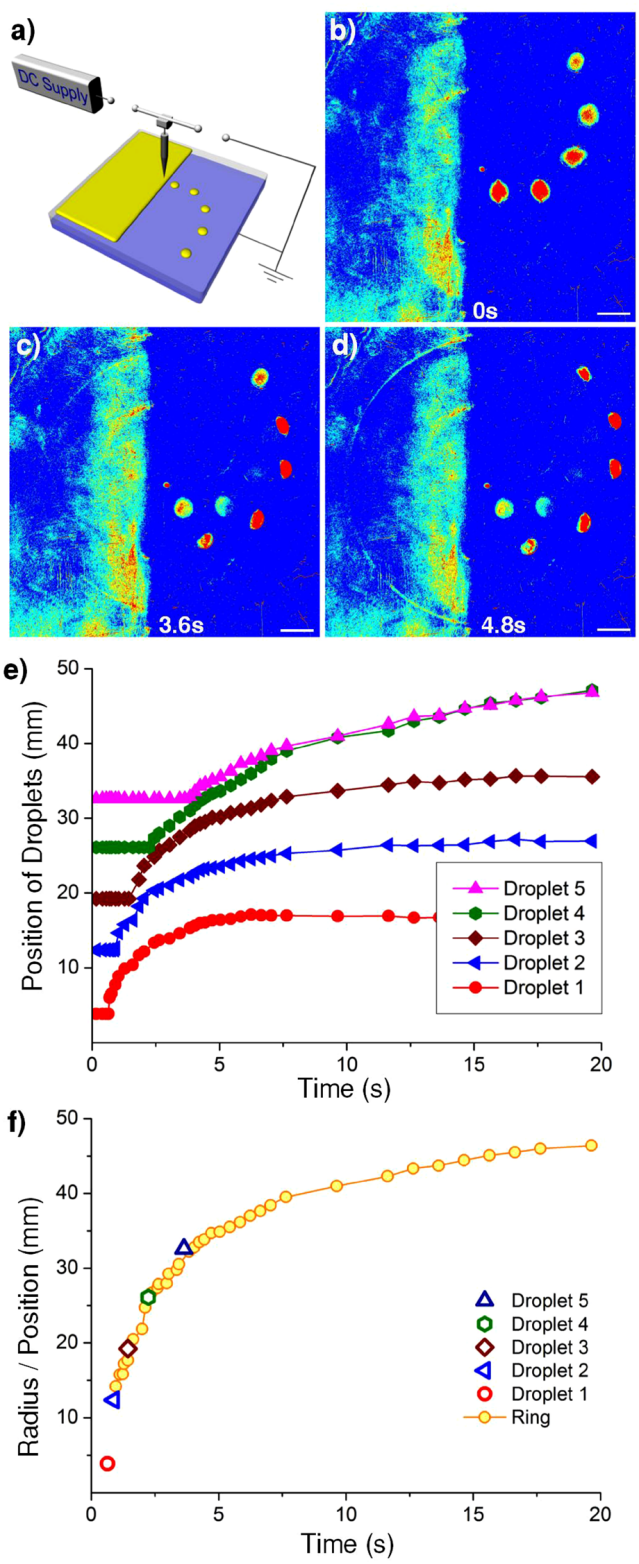
Retarded motion for discrete oil droplets vs. wave propagation on a continuous oil film. (**a**) Schematic setup for the experiments involving five oil droplets to the right and a half plane of an oil film with thickness of 0.05 mm to the left. We used a gap height of 15 mm, each droplet volume of 0.005 mL and placed from the epicenter at a distance of 7, 14, 21, 28 and 35 mm to the droplet center, or equivalently, at a distance of 3.9, 12.4, 19.2, 26.1 and 32.6 mm from the epicenter to the nearest droplet edge. A switch was used to slowly turn on the DC voltage bias from 0 kV at a speed of 2 kV/min to reach 9 kV so that five droplets remained motionless. Then after 5 s, the switch was shut off to initiate the shock wave propagation. (**b**–**d)** Photo images of half ring-shaped wave propagation on the oil film coated to the left of the substrate in comparison with the delayed motion for five individual oil droplets to the right. The reminiscent less bright images of the droplets are due to sedimentation of the fluorescence power even the droplets have moved away. **(e)** The time evolution for the position of those five droplets. (**f)** The time dependence of the radius for the half ring versus the time dependence of the position of each droplet when each started to move. The observed causality effect for delayed action on each droplet to move behaves wave-like propagation even though these droplets were not physically in contact. The error bar is too small to be displayed.

In contrast to presently known properties about the conventional solitons, the solitary shock waves we observed here are vastly different. To demonstrate these points, we have performed a series of experiments. As the first example, solitary wave propagation illustrated in Fig. 4a–d could occur regardless of whether one has a uniformly distributed oil layer or one has spatially disconnected oil droplets on a PET-ITO (polyethylene terephthalate) bilayer (see Supplementary Video 6). Each droplet began to move outward in sequence, starting from the droplet closest to the epicenter, resembles a wave-like action propagating outward. Such a peculiar behavior indicates that the driving force must come not from the oil surface tension itself but from the electric corona in the air. The time evolution for the half ring and the position of each droplet on the right are illustrated in Fig. 4c,f. These figures exhibit the time-retarded motion for each of the five droplets which were placed initially at various distances from the epicenter. The time for each droplet starting to move behaves like a delayed action as wave propagation, even though these droplets were not physically in contact, in contrast to ordinary surface waves that propagate only in a continuous medium.

## Discussion

In this letter we present observation of the formation and propagation of a single hair-thin solitary wave on an oil thin film. The wave propagation distance and speed at various DC voltage biases were measured. We have observed peculiar phenomena different from the ordinary waves, such as absence of wave interference and boundary reflection. In addition, we have observed wave-like time retarded action for individually oil droplets which were not physically in contact. All these behaviors strongly support that the driving force for the propagation of the ring-shaped wave front on oil is definitely not from the oil itself. We have found that a small objects hanging in the air could significantly affect the shape and the range of wave propagation. All evidences from our investigation indicate that Coulomb forces between the high voltage induced plasma, also known as electric corona, and the oil surface charges are the driving mechanism. Such solitary shock wave phenomena are shown to originate from mechanisms that differ from those for the conventional soliton or tsunami waves, and manifests entwined fluid dynamics and electro-dynamics of such a multi-phase medium through various observed unusual but interesting behaviors. We have used COMSOL to calculate the spatial charge distribution on oil due to air ionization. We have found the positive charges, in presence of voltage bias, are more concentrated at the epicenter at short times but the charge distribution becomes more even at longer time. Right after grounding of the needle the charges of the oil surface and the reminiscent charged air molecules with a slow neutralization rate could result an outward Coulomb force to induce the shock wave to propagate outward as observed in the experiments. Our observation of the oil fluid dynamics in the presence of high DC voltage could lead to some industrial applications such as water-oil separation, waste treatment and IC wafer processing. Etc. These extended work will be published elsewhere.

## Methods

### Materials and equipment

The following list contains the materials used in out experiments. PET film, thickness: 0.1 mm (DuPont Co., Ltd.); ITO glass (Wuhu Token Sciences Co. Ltd.), square-shaped 15 cm × 15 cm, thickness 1 mm, surface ITO film thickness 185 ± 2 nm, electric resistance: 8Ω; Steel needle: length 12 cm, diameter 0.59 mm; Fluoresce powder: YAG-04, Intematix Corp., particle size: 5–17 μm, excitation range 430–490 nm, emission peak 558 nm; Oliver oil: imported from Spain.

The following list contains the equipment used in this work. Video camera: ILCE-9 (SONY Corp.); Lens: 50 mmf/1.8 D Nikon; High voltage DC power: DW-P303-1ACF0 (Tianjin Dongwen High Voltage Power Supply Ltd.); Light source: KT-11B (Pvidi Co., Ltd.); Power switch: home built.

### Experimental setup

A PET film was placed horizontally on the conducting side of an ITO glass, and electrostatic attachment method was used to prepare a PET-ITO bilayer. A video camera was placed directly beneath the ITO substrate for recording, while a double goose-neck light source was placed above and on both sides of the substrate at a 45 degrees so that the illumination can be more even over the whole substrate. A steel needle was placed directly above the substrate and kept at a desired gap height. The arrangement of the needle, the substrate and the high voltage DC power supply is shown in Fig. 1a. Using a switch one can switch between the ON status with the needle connected to the power supply, versus the OFF status with disconnection between the needle and the power supply and simultaneous connection of the substrate and the needle to the electric ground.

### Experimental procedures

A mixture of oil and fluorescence powder at a ratio of 100:1 was prepared. After mixing by shaking for one minute in a test tube and waiting for 30 minutes the top portion of a mixture was extracted by a pipet. Coat an oil layer on the PET film, use a small nylon brush to make the oil film more evenly with a thickness of about 0.05 mm. Use a switch to turn on the high voltage DC power to a desired bias value. After the oil surface becomes stationary switch off the DC power to induce the shock wave on the oil film for video recording.

The schematic illustration for the setup for the experiment (Fig. 2g) involving two shock waves from a pair of parallel needles at a separation of 48 mm and above the polymer film of 15 mm. After the DC bias voltage reached 9 kV, then after 5 s the switch was shut off. Schematic setup illustration of the experiment (Fig. 2i) involving no reflection of the shock wave. A long narrow strip of a PET film of 120 mm × 9 mm × 0.1 mm was placed as a short wall at a distance from the epicenter of 23 mm, and then was glued onto the PET-coated substrate using a resin glue (EVA resin). The needle gap height is 15 mm ad the bias voltage was 9 kV.

Schematic setup for the experiment (Fig. 3a) involving an obstructing small acrylic object in the air. The small object has a dimension of 5 mm × 6 mm × 1.2 mm, placed at 12 mm away from the epicenter with a needle gap height of 15 mm. During the ON status this small object was placed at 20 mm above the surface, whereas during of OFF status before shutting off the switch two different heights of 4 and 10 mm were used for comparison for the small object above the surface. The bias voltage was 9 kV. Schematic setup diagram for the experiment (Fig. 3d) involving a tall vertical acrylic wall of a dimension of 110 mm × 50 mm × 1.2 mm. This wall is placed at 11 mm away from the needle. During the ON status the distance between the lower edge of this acrylic plate was placed at 20 mm above the polymer film surface, whereas during the OFF status before shutting off the power, the gap between the lower edge of this acrylic plate was changed to 4 and 10 mm, respectively, for comparison. The needle gap height was kept at 15 mm. The schematic setup diagram for the experiment (Fig. 3g) involving enclosure of the needle at the center by an acrylic cylinder of a length of 50 mm and an inner diameter of 30 mm and an outer diameter of 39 mm. During the ON status the tube was placed at 20 mm above the polymer film surface, whereas during of OFF status before shutting off the power, two different tube heights of 4 and 10 mm were used for comparison. The needle gap height was kept at 15 mm and the bias voltage was 9 kV.

Schematic setup for the experiment (Fig. 4a) involving five oil droplets to the right and a half plane of an oil film with thickness of 0.05 mm to the left, and a gap height of 15 mm, each droplet volume of 0.005 mL and placed from the epicenter at a distance of 7, 14, 21,28 and 35 mm, respectively. A switch was used to turn on the DC voltage bias from 0 kV slowly at a speed of 2 kV/min to reach 9 kV so that five droplets remained motionless. Then after 5 s, the switch was shut off to initiate the shock wave propagation and the droplets moving.

### Error analysis

The observed phenomena and the experimental results could be easily reproduced, and are not too sensitive to the temperature, humidity, composition and shape for the sharp steel needle. For the experiment represented by Fig. 1d, we have repeated the experiments five times, and we have waited over three hours for the residual charges on the oil to be neutralized to minimize accumulated undesirable results. The standard deviation and the coefficient of variation are tabulated in Table 1, showing good consistency with very small variations.

**Table 1:** The standard deviation and the coefficient of variation.

Voltage in each group	7 kV	8 kV	9 kV	10 kV	11 kV	12 kV
Standard Deviation	0.49	0.34	0.53	0.49	0.65	0.69
Coefficient of Variation	2.4%	0.99%	1.2%	1%	1.3%	1.2%

The error is greater for the readout of the position for the low-contrast wave front at earlier time is more difficult, whereas the error is smaller for the ring position at later times, we used the following approximated readout errors:

1 $${\rm{\varepsilon }}=\{\begin{array}{ll}4.8\,\exp (-0.069r)-0.4, & (r < 20)\\ 0.2, & (r < 20)\end{array}$$

where ε is the reading error and *r* the radius for the shock wave.

## Electronic supplementary material

Supplementary Video 1

Supplementary Video 2

Supplementary Video 3

Supplementary Video 4

Supplementary Video 5

Supplementary Video 6

Supplementary materal
