# Supplementary material for: Plasma-induced unconventional shock waves on oil surfaces
Source: Sci Rep. 2018 Dec 13;8:17813. doi: 10.1038/s41598-018-36278-3 (PMC6292859; doi:10.1038/s41598-018-36278-3)
Supplement: Supplementary file 7 — Supplementary materal [file 41598_2018_36278_MOESM7_ESM.docx]

Supplementary Materials for

**Plasma-induced unconventional shock waves on oil surfaces**

Guoliang Li^1†^, Ruiheng Hu^1†^, Jau Tang^1,2*^, Huai Zheng^3^ and Sheng Liu^1*^

^1^Institute of Technological Sciences, Wuhan University, Wuhan, Hubei 430072, China

^2^Arthur Amos Noyes Laboratory of Chemical Physics, California Institute of Technology, Pasadena, CA 91125, USA

^3^School of Power and Mechanical Engineering, Wuhan University, Wuhan, Hubei 430072, China

^†^These authors contributed equally to the work.

^*^To whom correspondence should be addressed: J. Tang: [jautang@caltech.edu](mailto:jautang@caltech.edu) and S. Liu: shengliu@whu.edu.cn

**This PDF file includes:**

Captions for Movies S1 to S6

**Supplementary Videos 1 to 6**

**Supplementary Video 1.** This 17 s movie recorded the image for the shock wave propagation which became slower as time increased. Fig. 1c was extracted from this movie and was color-coded for better visual effects.

**Supplementary Video 2.** This 9 s movie recorded propagation of two waves, each generated from each of the two needles. After collision of these two waves, a midway line boundary formed, in contrary to two colliding solitons where two wave fronts continue to move apart from the epicenters with no effects. Fig. 2b was extracted from this movie and was color-coded for better visual effects.

**Supplementary Video 3.** This 10 s movie recorded wave propagation after hitting a short acrylic wall with no reflection in contrast to conventional wave reflection at the boundary. Fig. 2d was extracted from this movie and was color-coded for better visual effects.

**Supplementary Video 4.** This 10 s movie recorded wave propagation with a vertically oriented acrylic plate on the left and above the surface. For a small gap height, the ring-shaped wave front on the left became an arc due to constraint of the electric corona in the air by the acrylic plate. Fig. 3b was extracted from this movie and was color-coded for better visual effects.

**Supplementary Video 5.** This 10 s movie recorded wave propagation due to the constraint of an acrylic cylindrical tube which was placed at 4 mm above the surface. The range of wave propagation became smaller due to this tube as compared the situation without the tube. Fig. 3e was extracted from this movie and was color-coded for better visual effects.

**Supplementary Video 6.** This 19 s movie recorded the wave propagation for the uniformly distributed oil film on the left resembles the time-retarded action of those five oil droplets on the right which were not physically connected. Figs. 4(b-d) were extracted from this movie and were color-coded for better visual effects.
